# Inflammation Regulation by Bacterial Molecular Patterns

**DOI:** 10.3390/biomedicines11010183

**Published:** 2023-01-11

**Authors:** Svetlana V. Guryanova, Anastasiya Kataeva

**Affiliations:** 1 Shemyakin-Ovchinnikov Institute of Bioorganic Chemistry of Russian Academy of Sciences, Ministry of Science and Higher Education of the Russian Federation, 117997 Moscow, Russia; 2 Medical Institute, Peoples’ Friendship University of Russia (RUDN University) of the Ministry of Science and Higher Education of the Russian Federation, 117198 Moscow, Russia; 3 Emanuel Institute of Biochemical Physics of RAS, 119334 Moscow, Russia

**Keywords:** innate immunity, innate immune memory, tolerance, TLR4, NOD2, LPS, lipopolysaccharide, muramyl peptide, GMDP, TNF-α, A20, ATF3

## Abstract

Stimulation of innate immunity by bacterial molecular patterns can induce an enhanced cellular immune response to pathogens that are associated with innate immune memory shaped by epigenetic changes. Immunological memory can be expressed in the acceleration/intensification of inflammation, as well as in the exact opposite—to maintain tolerance and non-response to a repeated stimulus. Tolerance is one of the central concepts of immunity and is ensured by the consistency of all parts of the immune response. The severe consequences of inflammation force researchers to study in detail all stages of the downstream pathways that are activated after exposure to a stimulus, while the formation of non-response to a pro-inflammatory stimulus has not yet received a detailed description. Elucidation of the mechanism of tolerance is an urgent task for the prevention and treatment of inflammatory diseases. The aim of this investigation was to study the dynamic changes in the gene expression of A20 and ATF3, the inflammation suppressors, against the background of the expression of the genes of the innate immunity receptors TLR4 and NOD2 and the pro-inflammatory cytokine TNF-α under the influence of TLR4 and NOD2 agonists, lipopolysaccharide (LPS) and glucosaminylmuramyl dipeptide (GMDP). The mechanism of inflammation regulation by bioregulators of bacterial origin—LPS and GMDP—was evaluated in vitro in human peripheral blood mononuclear cells and in vivo after i.p. administration of LPS and GMDP to mice. Gene expression was assessed by RT-PCR. Innate immune receptors and the pro-inflammatory cytokine TNF-α were found to develop early in response to LPS and GMDP, both in vitro and in vivo. Genes of cytosolic proteins controlling inflammation (A20 and ATF3) were expressed later. Prior exposure of the innate immune system to LPS and muramyl peptides may modulate host defense against acute inflammation. As a result of the study, new data were obtained on dynamic changes in deubiquitinase A20 and the transcription factor ATF3, which are involved in the limitation and suppression of inflammatory reactions caused by fragments of bacterial cell walls—LPS and GMDP. Thus, bioregulators of bacterial origin LPS and GMDP, along with pro-inflammatory factors, activate the expression of genes that suppress inflammation, which should be considered when analyzing data from studies of the pro-inflammatory properties of LPS and GMDP and when developing drugs based on them.

## 1. Introduction

Maintaining the homeostasis of the human body in the environment of microorganisms is provided by numerous coordinated processes, including the stages of recognition of pathogenicity patterns (pathogen-associated molecular patterns, PAMPs), response, and tolerance formation. Recognition is carried out by innate immunity receptors TLR, NLR, CLR, and RLR, with a fairly high degree of homology in different species of organisms, recognizing not only PAMPs of viruses, fungi, and protozoa but also their metabolic products [1,2,3]. The response is implemented through protein-protein interactions, a cascade of further signaling events leading to a change in the spectrum of proteins on the membrane surface, released polypeptides, mediators, and metabolites. The result is a change in the functions of a cell. At the same time, each immunocompetent cell and each mucosal cell are exposed to various microorganisms, while various PAMPs act on innate immunity receptors belonging to different classes. For an adequate response, strict coordination of the speed, intensity, and type of response must be observed.

It was found that the response rate of innate immunity can be markedly reduced upon repeated exposure [4,5,6]. This observation led to the introduction of the term “innate immune memory” [7,8]. The mechanism of innate immune memory is defined at the level of epigenetic changes in chromatin modifications: methylation/demethylation and acetylation/deacetylation of DNA and histones and is accompanied by the synthesis and degradation of the corresponding inhibitors, regulators, transcription factors, and small RNAs. At the same time, innate immune memory can be considered more broadly, meaning not only an increase in the rate of repeated exposure to a stimulus but also its decrease, up to a complete lack of response and the formation of tolerance [8].

Tolerance is one of the central concepts of the immune response and is considered a protective strategy against infectious diseases [9]. Identification of the mechanisms of tolerance formation will provide new approaches to the treatment of infections and other diseases and drug design.

Currently, with the discovery of the influence of bacterial fragments on epigenetic changes, new questions have arisen that require explanation. In particular, what exactly determines the rapid reactivity of innate immunity to a repeated stimulus, and what determines the formation of tolerance? The accumulated empirical material testified both to the formation of hyperreactivity to repeated stimuli [5,6,7] and non-response [9,10,11]. For example, lipopolysaccharide (LPS) priming four days rather than two days prior to infection enhanced bacterial clearance and promoted survival in mice with septic peritonitis [12]. The mechanism underlying the protective effect of LPS, administered four days before LPS-induced inflammation, involves the production of anti-inflammatory factors with a significant time delay, which, based on the study, took four days starting from the moment of LPS administration. The exact answer to the question about the negative regulation of inflammation by LPS requires investigation. A similar positive effect was found for GMDP in a model of septic inflammation. The administration of glucosaminyl muramyl dipeptides (GMDP) and its analogs to mice 14 days before the lethal dose of LPS resulted in 70–90% animal survival [13]. At the same time, the combined administration of LPS and muramyl peptides showed a synergistic effect and accelerated the death of the animals [12,13]. Prolonged exposure to muramyl peptides in human macrophages suppressed the synthesis of pro-inflammatory cytokines TNF-alpha, IL-8, and IL-1beta [14]. Thus, prior exposure of the innate immune system to LPS and muramyl peptides may modulate host defense against acute inflammation.

LPS is a component of the cell walls of Gram-negative bacteria. Muramyl peptides (MP) and glucosaminyl muramyl dipeptides are part of the peptidoglycans of Gram-negative and Gram-positive bacteria that inhabit the gastrointestinal tract and human skin. LPS and GMDP are not synthesized in eukaryotic organisms. Normally, they are formed during the degradation of the microflora and the action of host enzymes on the mucosal surfaces [15,16], as well as under the action of the bacteria’s own enzymes (hydrolases, peptidases, amidases), which disintegrate the bacteria cell wall for growth, reproduction [17,18] and competition for habitat [19].

LPS and MP are the most studied of all PAMPs due to their importance in the regulation of inflammatory processes in all human organs and systems [20,21,22,23,24]. Numerous experiments on laboratory animals are aimed at studying the pro-inflammatory properties of LPS [25,26,27], while the anti-inflammatory properties of low doses of LPS are rarely the subject of research, despite the obvious fact of regular human exposure to LPS, which originates from the endogenous microflora. Nevertheless, there are data on the protective effect of LPS. For example, in a model of chronic spinal cord injury, systemic injection of a low dose of LPS increased the effectiveness of rehabilitation training on the function of the forelimbs of rats [28], but no explanation was given for the reasons for the effectiveness. The importance of a comprehensive study of the effect of LPS is crucial due to the recent use of LPS as a model inflammatory agent in human studies [29,30].

In our previous studies, a double effect of LPS and GMDP was revealed—a significant increase in the inflammatory process and mitigation of inflammation in the model of septic shock and experimental bronchial asthma [13,31]. It was shown that an MP-based drug changes the phenotypic and functional characteristics of immunocompetent cells [20,32], contributing to anti-infective protection against bacterial and viral pathogens and reducing the severity of inflammatory processes [32,33,34]. The receptors that recognize LPS are TLR4, located on the cell surface. Muramyl peptides are recognized by cytoplasmic NOD1 and NOD2 receptors belonging to the NLR family. Muramyl peptides formed under the action of lysozyme, present on human mucous surfaces, are represented by disaccharide-containing derivatives, glucosaminyl muramyl peptides, whose sensor is the NOD2 receptor [35,36]. Downstream signals after exposure to TLR4 and NOD2 receptors at a certain stage converge to the activation of the transcription factor NFkB, and its translocation to the nucleus occurs; as a result, pro-inflammatory cytokines and mediators are synthesized [37,38,39]. Synthesized proinflammatory factors can have a positive effect, for example, restoring insufficient Th1 activity, activating neutrophil granulocytes and cytotoxic T lymphocytes, thus replenishing their impaired functions [20], or compensating for impaired functions of innate immunity, for example, in agammaglobulinemia [40]. Pro-inflammatory factors in the context of developing inflammation have a negative effect, for instance, in allergic inflammation in an experimental model of bronchial asthma [31]. We found that the combined administration of LPS and GMDP, together with the allergen, aggravated the severity of the inflammatory process and increased eosinophilia and neutrophilia. However, early long-term exposure to TLR4 and NOD2 six days before the first allergen exposure, LPS and GMDP reduced lung infiltration, eosinophilia, and neutrophilia and mitigated the degree of allergic inflammation [31]. The purpose of this study was to explain the mechanism of the previously discovered dual effect of LPS and GMDP—the enhancement or mitigation of inflammation—at the level of regulators of intracellular processes—deubiquitinase A20 and activating transcription factor 3 (ATF3), while TNF-α was used as a marker of inflammation.

## 2. Materials and Methods

### 2.1. Animal Care

Male 4-week-old BALB/C mice were purchased from Pushchino (Russia) and maintained under standard conditions with a 12-h dark/light cycle under defined pathogen-free conditions. Animals were housed in polypropylene cages and had free access to food and water. All experiments were performed in accordance with the Geneva Convention “International Guiding Principles for Biomedical Involving Animals” (Geneva, 1990), as well as the Declaration of Helsinki by the World Medical Association on the humane treatment of animals (2000 revision). The study was approved by the Ethics Committee of the Peoples’ Friendship University of Russia (Moscow, Russia).

### 2.2. Sample Administration to Mice

Mice were randomly divided into two groups of 15 animals in each group.

In the first group of mice, on days 1, 2, 3, 4, and 5, an intraperitoneal injection (ip) of 1 μg/animal of LPS (Ultra-pure, Invivogen, San Diego, CA, USA) in 0.2 mL of sterile saline (PBS) was made. On days 0, 1, 6, 11, and 21, blood samples were collected.

In the second group, intraperitoneal injections of 5 μg/animal of GMDP (JSC Peptek, Russia) were administrated on days 1, 2, 3, 4, and 5 in 0.2 mL of PBS. On days 0, 1, 6, 11, and 21, blood samples were collected.

### 2.3. Isolation of Mononuclear Cells

Blood samples were taken from the heart of mice after 0, 2, 6, 11, and 21 days. Blood was collected in tubes (Vacuette, Greiner Bio-One, Kremsmünster Austria) with anticoagulant (0.1 mL of 2.7% EDTA salt solution; (pH 7.2–7.4) per 1 mL of blood). All procedures were performed under sterile conditions at room temperature. Whole blood was diluted 1:3 with phosphate-buffered saline (PBS), layered on cell separation media Lympholyte CL 5015 (Cedarlane Laboratories Limited, Burlington, ON, Canada), and centrifuged for 40 min at 400× *g*. Mononuclear cells (MNCs) were washed twice (10 min; 1000 rpm) by centrifugation in excess PBS and resuspended in complete medium RPMI 1640 containing 10% fetal bovine serum, 100 U/mL penicillin, 100 μg/mL streptomycin and 10 mM Hepes buffer, pH 7.2 (Life Technologies, Carlsbad, CA, USA). Cell viability was determined by trypan blue staining. 

### 2.4. Ethics Approvement and Voluntary Consent for Personal Data Processing and Publishing

The study protocol was approved by the Ethics Committee of the Peoples’ Friendship University of Russia (protocol code N11S/20, 15 December 2020). The sampling methods in patients were performed in accordance with the Operational Guidelines for Ethics Committees That Review Biomedical Research (https://apps.who.int/iris/handle/10665/66429, access date 12 November 2022). Voluntary written informed consent was obtained from all healthy volunteers involved in the study with an agreement to take part in the study and personal data processing for publishing this paper.

### 2.5. In Vitro Studies

For human PBMC isolation, whole blood (7 mL) from healthy volunteers (22–23 years old) was collected in tubes (Vacuette, Greiner Bio-One, Kremsmünster, Austria) with anticoagulant (0.1 mL of 2.7% EDTA salt solution; (pH 7.2–7.4) per 1 mL of blood). All procedures were performed under sterile conditions at room temperature. Whole blood was diluted 1:3 with phosphate-buffered saline (PBS), layered on cell separation medium Lympholyte CL 5015 (Cedarlane Laboratories Limited, Burlington, ON, Canada), and centrifuged for 40 min at 400× *g*. Mononuclear cells (MNCs) were washed twice (10 min; 1000 rpm) by centrifugation in excess PBS and resuspended in complete medium RPMI 1640 containing 10% fetal bovine serum, 100 U/mL penicillin, 100 μg/mL streptomycin and 10 mM Hepes buffer, pH 7.2. Cell viability was determined by trypan blue staining. Mononuclear cells were cultured for 24 h in the presence of LPS (1 μg/mL) or GMDP (5 μg/mL) or without agonists. Then the medium was removed, and cell lysates were collected for analysis of gene expression after 30 min, 1, 2, 4, 12, and 24 h.

### 2.6. Quantitative RT-PCR

The study of gene expression was performed using real-time reverse transcription polymerase chain reaction (RT-PCR-RT). The expression of the A20, ATF3, NOD2, TLR4, TNF-α, and GAPDH genes was studied in mononuclear cells of mice and from three healthy donors. Total RNA was isolated from cells using the TRIzol^®^ reagent (Invitrogen, Carlsbad, CA, USA) according to the manufacturer’s protocol. To synthesize cDNA, a reverse transcription reaction was performed using the Mint enzyme (Evrogen, Moscow, Russia) according to the manufacturer’s standard protocol. The polymerase chain reaction was carried out in a CX96 thermal cycler (BioRad, Hercules, CA, USA) using a qPCRmix-HS SYBR reaction mixture (Evrogen, Moscow, Russia). The amplification protocol included: 1 cycle of 10 min denaturation at 94 °C, 38 cycles of 20 sec at 94 °C for denaturation of DNA strands, 20 sec at 60 °C for annealing of primers with template) and 40 sec at 72 °C for the synthesis of complementary DNA strands, and 1 cycle of final synthesis for 3 min at 72 °C. The calculation of the relative level of mRNA of the target genes was performed using the 2^−ΔΔCt^ method. The relative substrate concentration was normalized to the average GAPDH amplification data and the expression of this gene in the control sample (unstimulated cells). All reactions were performed in triplicate and included negative controls. Relative expression differences of more than 2 times were considered significant. Primers used in RT PCR analysis are represented in Supplementary Materials.

### 2.7. Statistical Analysis

Statistical data processing was carried out using Prism 8.0.2. Medians were used as estimates of the studied parameters due to the non-correspondence of the samples to the normal distribution. The variability of the central trends was expressed by 95% confidence intervals. The obtained values were compared using the nonparametric Mann–Whitney test and the Wilcoxon rank-sign test with Bonferroni correction for multiple comparisons. Differences were considered statistically significant with *p* < 0.05. 

## 3. Results

Our previous studies have found a dual effect of LPS and GMDP—an increase in inflammation when co-administered with an antigen—and a decrease in inflammation when administered with LPS and GMDP in advance of the antigen [31]. In order to explain the mitigation of inflammation, analysis of the expression of regulatory elements was implemented at three levels of signaling pathways, which were carried out: (a) at the level of receptors; (b) at the level of transcription factors, and ubiquitinylation suppressor; (c) at the level of expression of pro-inflammatory cytokines in vivo and in vitro.

**Figure 1:**
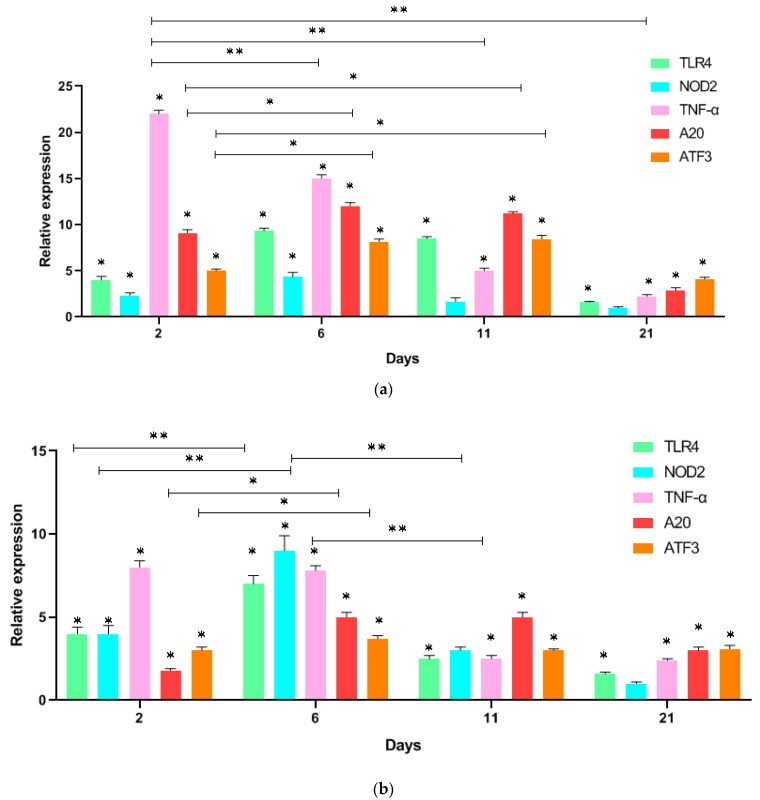
Relative expression of genes TLR4, NOD2, TNF-α, A20, and ATF3 with normalization for expression of GAPDH before i.p. stimulation (day 1) in mononuclear cells of mice on the 2, 6, 11, 21 days; (**a**) ip injection of LPS; (**b**) ip injection of GMDP; relative expression differences of more than 2 times were considered significant; data are represented as an average of three independent samples and error bars represent standard deviation; * *p* < 0.01; ** *p* <0.001.

Introduction of i.p. LPS to mice 24 h after the first administration of LPS in the blood serum of mice significantly increased the expression of all the studied genes. TNF-α after 24 h of LPS administration showed the highest values 21.9-fold (21.9 ± 0.9), (*p* = 0.001), which decreased at 6, 11, and 21 days. At the same time, the maximum values of 12.1 times (12.1 ± 0.4), (*p* = 0.01) A20 reach on day 6, on day 11, they retain high values 11 times (11.0 ± 0.35), (*p* = 0.01), and on day 21 these values already slightly exceed the level of TNF-α expression, but this difference is not statistically significant (Figure 1). At the same time, high levels of 9.1 (9.1 ± 0.3), (*p* = 0.01) and 8.7 times (8.7 ± 0.25), difference (*p* = 0.01) of TLR4 expression persist on days 6 and 11.

As a result of exposure to LPS, an increase in the expression of the NOD2 receptor was also observed, the maximum value of which was 4.4 times (4.4 ± 0.2), (*p* = 0.01); which took place on the 6th day, but by the 11th day the values decreased to 1.5 (1.5 ± 0.15), not significant (*p* > 0.05), and by the 21st day already returned to the initial values. The expression of deubiquitinase A20 increased from the first day by 9.1 times (9.1 ± 0.4), (*p* = 0.01) and remained high for 11 days, and on day 21, its expression was increased by 3 times (3.0 ± 0.16), (*p* = 0.01) compared to the initial value. Activating transcription factor 3 significantly increased within 24 h up to 4.9 times (4.9 ± 0.2), (*p* = 0.01), and on the 6th day, the values reached a maximum of 12.1 times (12.1 ± 0.4), (*p* = 0.01), and high values of its expression by 4.1 times (4.1 ± 0.2), (*p* = 0.01) persisted until the 21st day.

Analysis of the obtained data shows that the administration of LPS to mice significantly increases the expression of both TLR4 and NOD2 receptors, as far as TNF-α. In addition, the expression of deubiquitinase A20 also immediately increases. The expression of ATF3 was delayed but maintained high values for 11 days after injection. 

The administration of GMDP to mice stimulated gene expression to a much lesser extent than LPS, but the level of NOD2 increased already on the first day by up to 4.3 times (4.3 ± 0.2), (*p* = 0.01), similar to TLR4 which increased by 4.2 times (4.2 ± 0.2), (*p* = 0.01), and their maximum values were also observed by the 6th day to increase by up to 8.7 times (8.7 ± 0.35), (*p* = 0.01) for TLR4 and by 9.5 times (9.5 ± 0.35), (*p* = 0.01) for NOD2. An increase in A20 expression by 5.1 times (5.1 ± 0.15), (*p* = 0.01) was observed only by the 6th day and remained increased for 11 days after injection. Interestingly, GMDP stimulated the expression of not only its own receptor (NOD2) but also of TLR4.

These data show that the maximum values and expression of receptors (TLR4, NOD2) and TNF-α, as well as the transcription factor ATF3 and deubiquitinase A20, reach maximum values by the 6th day, after which the expression of TLR4, NOD2, and TNF-α noticeably decreases, and the expression of A20 and ATF3 is still elevated (Figure 1). 

**Figure 2:**
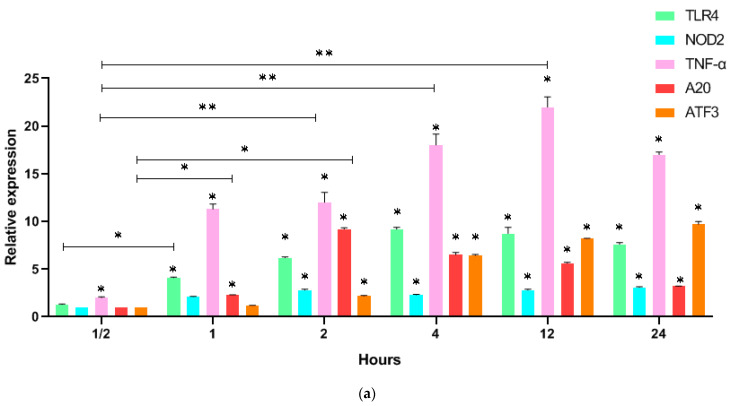

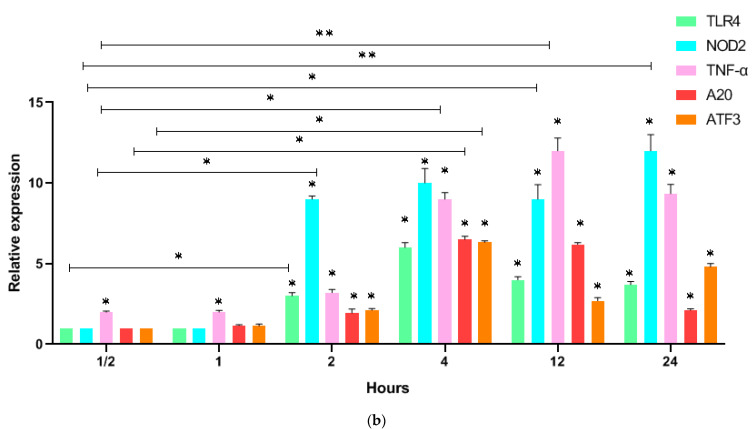
Relative expression of genes TLR4, NOD2, TNF-α, A20, and ATF3 with normalization for expression of GAPDH before in vitro stimulation in mononuclear cells of healthy donors after 30 min, 1, 2, 4, 12, and 24 h: (**a**) LPS; (**b**) GMDP; relative expression differences of more than 2 times were considered significant; data are represented as an average of three independent samples and error bars represent standard deviation; * *p* < 0.01; ** *p* < 0.001.

In vitro studies showed that LPS stimulation of human mononuclear cells for 30 min leads to an increase in TNF-α by 2 times (2.0 ± 0.06), (*p* = 0.01), after an hour—by 11 times (11.1 ± 0.3), (*p* = 0.001), after an hour—by 12 times (12.2 ± 0.4), (*p* = 0.001), and by 12 h reaches a maximum of 22 times (22.1 ± 0.5), (*p* = 0.001) (Figure 2). Expression of the intrinsic receptor (TLR4) increases 4-fold (4.2 ± 0.2), (*p* = 0.01) after an hour, reaches a maximum of 9-fold (9.2 ± 0.4), (*p* = 0.01) after 4 h and remains at this level up for to 12 h, and remains elevated 7.5-fold (7.5 ± 0.25), (*p* = 0.01) for up to 24 h. An increase in TNF-α expression by 2 times (2.05 ± 0.15), (*p* = 0.01) is recorded after 30 min, reaches a maximum of 22.1 times (21.1 ± 0.95), (*p* = 0.001) values by 12 h, and then decreases. The expression of regulatory factors—A20 and ATF3 by 30 min remains unchanged and increases much more slowly than TNF-α. The maximum value of 9.2 (9.2 ± 0.45), (*p* = 0.01) A20 reaches 2 h, and ATF3 reaches 9.7 (9.7 ± 0.25), (*p* = 0.01) times 24 h after stimulation. Thus, A20 and ATF3 are expressed later than TNF-A and TLR4 after LPS stimulation in vitro.

When human mononuclear cells are stimulated with GMDP, only TNF-α expression significantly increases by 2.2 times (2.1 ± 0.1), (*p* = 0.01) after 30 min, TLR4 expression increases by 3.2 times (3.2 ± 0.15), (*p* = 0.01) after 2 h, reaches a maximum value of 6.1 times (6.1 ± 0.3), (*p* = 0.01) by 4 h, then decreases within 24 h up by 3.7 times (3.7 ± 0.3), (*p* = 0.01), (Figure 2). Expression of the NOD2 receptor remains unchanged within an hour, increases 9-fold (9.05 ± 0.35), (*p* = 0.01) after 2 h, and remains elevated for 24 h. TNF-α increases from the first minutes after stimulation, reaching a maximum value of increasing by 12.1 times (12.1 ± 0.4), (*p* = 0.001) after 12 h and maintaining high values for 24 h. A20 begins to increase 2 h after stimulation and reaches its maximum values of increasing by 6.5 times (6.5 ± 0.3), (*p* = 0.01) after 4 h, and high values of increasing by 6.2 times (6.2 ± 0.3), (*p* = 0.01) persist for 12 h and decrease by 2.2 times (2.2 ± 0.1), (*p* = 0.01) after 24 h. The expression of ATF3 increases much more slowly; only after 2 h it rises by 2.1 times (2.1 ± 0.1), (*p* = 0.01), the maximum values of increasing by 6.3 times (6.3 ± 0.3), (*p* = 0.01) take place after 4 h, and an increased level of expression remains for 24 h.

## 4. Discussion

Previously, we showed that bioregulators of bacterial origin, LPS and GMDP, have a dual effect—they can increase or alleviate inflammation [31]. Moreover, this effect is manifested both at the cellular and humoral levels. In an experimental model of bronchial asthma, the combination of LPS and GMDP with the allergen contributed to the infiltration of the lungs by macrophages, lymphocytes, neutrophils, and eosinophils, as well as a significant increase in IgG2a. Preliminary multiple administration of LPS or GMDP reduced lung infiltration by reducing neutrophils and eosinophils and also reduced IgA and IgE levels. To explain the mechanism of action of LPS and GMDP on the regulation of allergic inflammation, this study analyzed the genes expression of cytosolic proteins A20 and ATF3, responsible for the suppression of inflammation, in comparison with TNF-α, LPS, and NOD2 genes. As a result of research, it was shown that when BBO is exposed to mononuclear cells for 60–120 min, the genes of innate immunity receptors TLR4 and NOD2, as well as TNF-α, are activated. It is known that TNF-α has a powerful pro-inflammatory stimulating effect and induces the expression of NOD1, NOD2, and TNF mRNA [41,42,43,44,45]. Interestingly, the NOD1 receptor, which recognizes muropeptides of Gram-negative bacteria with a mesodiaminopimelic acid residue (meso-DAP), can compete with NOD2 for binding to its ligand in human monocytes and activate additional downstream pathways [46]. The observed stimulation under the action of GMDP of the expression not only of its own receptor (NOD2) but also of TLR4 can serve as an explanation for the synergism of GMDP and LPS when they are used together [13,31].

Thus, at the initial stage of the inflammatory process, prerequisites are formed for the formation of a positive feedback loop that enhances inflammation in several directions at once—an increase in the expression of receptors responsible for stimulus perception and an increase in the expression of TNF-α, an inflammatory cytokine. It is known that TNF-α activates leukocytes, stimulates the production of other pro-inflammatory cytokines—IL-1, IL-6, and IL-8, and is also a pleiotropic messenger in the regulation of metabolic processes [47,48]. 

At an early stage of the inflammatory process, mRNA (30–60 min) A20 and ATF3 are not detected and appear after 2–4 h. Activating transcription factor 3 is a member of the transcription factor family of proteins that bind activating transcription factor/cAMP. ATF-3 is induced under physiological stress in various tissues and can act as a transcriptional suppressor or activator in various tissues, forming homodimers or heterodimers with other members of the ATF/CREB family [49,50]. In macrophages, ATF3 can act as a negative regulator of inflammation by binding to the p65 subunit of NFkB [51,52]. Deubiquitinase A20 removes ubiquitin from ubiquitinated substrates and thereby alters the activation of downstream regulatory proteins, reversing the process. A20 is required for normal NFκB signaling and suppression of inflammation [53]. 

**Figure 3:**
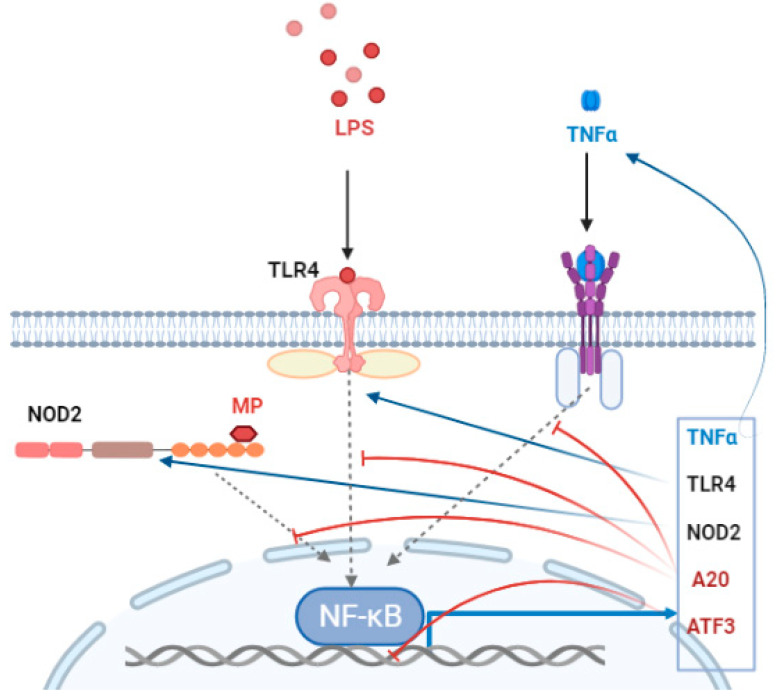
Positive and negative feedback during lipopolysaccharide (LPS) and muramyl peptide (MP)—induced inflammation. Blue arrows—activation of inflammation; red lines—suppression of inflammation.

In our study, it was found for the first time that with prolonged exposure to GMDP, the anti-inflammatory factors which are inhibiting inflammation, A20 and ATF3, are activated. At the same time, the maximum values of A20 and ATF3 mRNA persist for a longer time than TLR4, NOD2, and TNF-α mRNA. With an increase in the concentration of A20 and ATF3, a negative feedback loop is formed that stops inflammation (Figure 3).

The limitations of this study include a small number of studied cytokines and transcription factors. In addition, studies conducted in vivo in rodents should be extrapolated to humans with caution since many activation pathways in rodents are different from those in humans, including the regulation of TLR receptor expression [54]. These differences should be considered in connection with the increasing number of studies on the effects of LPS and muramyl peptides in humans.

## 5. Conclusions

Thus, the protective effect of BBO observed in our studies in models of septic shock, allergic inflammation, as well as in infectious diseases [13,20,31,32] can be explained by the appearance of anti-inflammatory factors during stimulation, including regulatory enzymes and transcription factors that limit both the synthesis of innate immunity receptors and the expression of cytokines.

It should be noted that the activation of TLR2 and NOD2 receptors can also have non-canonical descending pathways that do not involve NFkB and corresponding inhibitors [55,56]. During inflammation, a cascade of pro-inflammatory cytokines induces many processes, including the expression of its own receptors and regulators of all downstream pathways. Due to the involvement of hundreds of proteins in the inflammatory process, a systematic approach with visualization of relationships is needed [57,58]. To manage the inflammatory process, it is also necessary to consider the individual characteristics of the organism, including analysis of the genome, transcriptome, proteome, metabolome, and microbiome. Only with an integrated approach is it possible to form predictive models of personalized medicine and develop effective methods of therapy and prevention.

## Data Availability

Not applicable.

## Supplementary Materials

The following supporting information can be downloaded at: https://www.mdpi.com/article/10.3390/biomedicines11010183/s1, Table S1. Primers used in RT PCR analysis. References [59,60,61,62,63,64,65,66] are cited in the Supplementary Materials.
