# Supplementary material for: Inflammation Regulation by Bacterial Molecular Patterns
Source: Biomedicines. 2023 Jan 11;11(1):183. doi: 10.3390/biomedicines11010183 (PMC9855958; doi:10.3390/biomedicines11010183)
Supplement: Supplementary file 1 [file biomedicines-11-00183-s001.zip › biomedicines-2102085-supplementary.pdf]

biomedicines

MDPI

Article

Inflammation Regulation by Bacterial Molecular Patterns

Svetlana V. Guryanova[1, 2*] and Anastasiya Kataeva[3]

[1] Shemyakin-Ovchinnikov Institute of Bioorganic Chemistry of Russian Academy of Sciences, Ministry of Science and Higher Education of the Russian Federation, 117997 Moscow, Russian Federation
[2] Peoples' Friendship University of Russia (RUDN University) of the Ministry of Science and Higher Education of the Russian Federation, Medical Institute, 117198 Moscow, Russian Federation
[3]Emanuel Institute of Biochemical Physics of RAS, 119334 Moscow, Russian Federation
* Correspondence: svgur@ibch.ru; Tel.: +79153150073

Supplementary Materials

Table S1. Primers used in RT PCR analysis.

Genes	Forward primer	Reverse primer	t	Product size	Ref.
		Human			
A20	5′ -GGACTTTGCGAAAGGATCG-3′	5′ -TCACAGCTTTCCGCATATTG-3′	57	130	[1]
ATF3	5' -CATCTTTGCCTCAACTCCAG-3′	5' -GACACTGCTGCCTGAATCCT-3′	59	147	[2]
NOD2	5′-GCCACGGTGAAAGCGAAT-3′	5′-GGAAGCGAGACTGAGCAGACA -3′	60	148	[3]
TLR4	5' -TGGGCAACCTGCTCTACCTA-3′	5' -GCTGTAGCTCGTTGGCAGA-3′	60	117	[4]
TNF-α	5′ -CCCAGGCAGTCAGATCATCTTC-3′	5′ -AGCTGCCCCTCAGCTTGA-3′	64	85	[3,5]
GAPDH	5′ - TGCACCACCAACTGCTTA -3′	5′ - GGATGCAGGGATGATGTT -3′	54	177	[6]
		Murine			
A20	5′ -AAGCTCGTGGCTCTGAAAAC-3′	5′ -TTCCTCAGGACCAGGTCAGT-3′	60	101	[1]
ATF3	5′- GAAGATGAGAGGAAAAGGAGGCG-3′	5′-GCTCAGCATTCACACTCTCCAG-3′	60	130	[7]
NOD2	5' -CGACATCTCCCACAGAGTTGTAATCC-3'	5' -GGCACCTGAAGTTGACATTTTGC-3′	65	123	[8]
TLR4	5′- TTCAGCACTC TACTTTAAAA-3′	5′- TAATAATTGAAGTCTATGGAGGG-3′	53	290	[2]
TNF-α	5′ -GTGGAACTGGCAGAAGAGGC-3′	5′ -AGACAGAAGAGCGTGGTGGC-3′	62	122	[3]
GAPDH	5′ -CGTCCCGTAGACAAAATGGT-3′	5′ -TTGATGGCAACAATCTCCAC-3′	57	226	[1]

Note: A20, is a zinc finger protein identified as a negative regulator of NFκB activity; ATF3, Activating transcription factor 3; NOD2, Nucleotide-binding oligomerization domain-containing protein 2; TLR4, Toll-like receptor 4; TNF-α, tumor necrosis factor α; GAPDH, glyceraldehyde-3-phosphate dehydrogenase

1. Günthner R, Kumar VR, Lorenz G, Anders HJ, Lech M. Pattern-recognition receptor signaling regulator mRNA expression in humans and mice, and in transient inflammation or progressive fibrosis. Int J Mol Sci. 2013 Sep 4;14(9):18124-47. doi: 10.3390/ijms140918124.
2. Primer-BLAST. https://www.ncbi.nlm.nih.gov/tools/primer-blast/
3. King AE, Horne AW, Hombach-Klonisch S, Mason JI, Critchley HO. Differential expression and regulation of nuclear oligomerization domain proteins NOD1 and NOD2 in human endometrium: a potential role in

innate immune protection and menstruation. Mol Hum Reprod. 2009 May;15(5):311-9. doi: 10.1093/molehr/gap020.
4. Wan J, Shan Y, Fan Y, Fan C, Chen S, Sun J, Zhu L, Qin L, Yu M, Lin Z. NF-κB inhibition attenuates LPS-induced TLR4 activation in monocyte cells. Mol Med Rep. 2016 Nov;14(5):4505-4510. doi: 10.3892/mmr.2016.5825.
5. Hassan-Nejhad M, Bagheri M, Khadem-Vatani K, Seyed Mohammad Zad MH, Abdi Rad I, Rahimi B, Rostamzadeh A, Rahimlou A. Tumor Necrosis Factor-alpha Gene Expression in PBMCs of Iranian Azeri Turkish Patients with Premature Coronary Artery Disease (Age .50 Years). Maedica (Bucur). 2018 Mar;13(1):12-16. PMID: 29868134; PMCID: PMC5972780.
6. Kim HJ, Yoon HM, Kwon O, Lee WJ. The Effect of Pueraria Lobata/Rehmannia Glutinosa and Exercise on Fatty Acid Transporters Expression in Ovariectomized Rats Skeletal Muscles. J Exerc Nutrition Biochem. 2016 Sep;20(3):32-38. doi: 10.20463/jenb.2016.09.20.3.5
7. https://www.origene.com/catalog/gene-expression/qpcr-primer-pairs/mp200254/atf3-mouse-qpcr-primer-pair-nm_007498
8. Bowcutt R, Bramhall M, Logunova L, Wilson J, Booth C, Carding SR, Grencis R, Cruickshank S. A role for the pattern recognition receptor Nod2 in promoting recruitment of CD103+ dendritic cells to the colon in response to Trichuris muris infection. Mucosal Immunol. 2014 Sep;7(5):1094-105. doi: 10.1038/mi.2013.125.
